# Intracellular Alpha-Synuclein and Immune Cell Function

**DOI:** 10.3389/fcell.2020.562692

**Published:** 2020-10-15

**Authors:** Veselin Grozdanov, Karin M. Danzer

**Affiliations:** Department of Neurology, Ulm University, Ulm, Germany

**Keywords:** alpha-synuclein, Parkinson’s disease, immune cell function, microglia, monocytes

## Abstract

Intracellular alpha-synuclein has numerous effects on different functions of the cell. Although it is expressed in a wide spectrum of cell types from different lineages, most of our knowledge about it was generated by studying neuronal or glial cells. However, the role of immune cells in Parkinson’s disease and related synucleinopathies has recently emerged. Altered immune cell phenotypes and functions have been reported not only in animal models, but also in human disease. While the response of immune cells to extracellular alpha-synuclein has been thoroughly studied, insights into the effects of endogenously expressed or taken-up alpha-synuclein on the function of immune cells remain scarce. Such insights may prove to be important for understanding the complex cellular and molecular events resulting in neurodegeneration and aid the development of novel therapies. We review the current state of knowledge about how alpha-synuclein and its pathologic manifestations affect the phenotype and function of peripheral and central nervous system (CNS) immune cells, and discuss the potential of this topic for advancing our understanding of synucleinopathies.

## Introduction

**FIGURE 1:**
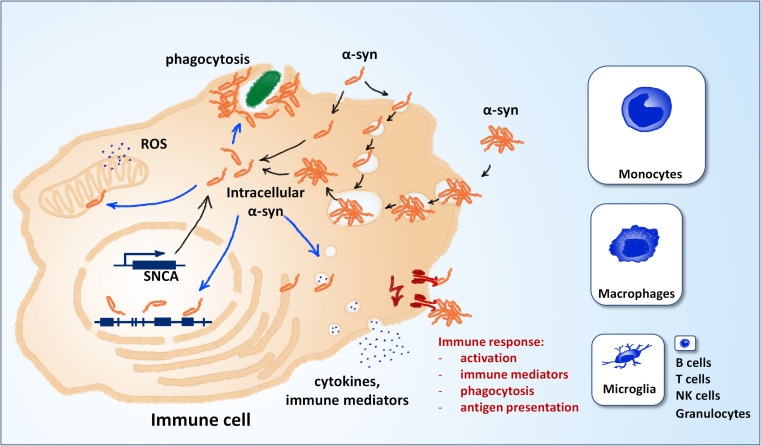
Alpha-synuclein and immune cell function. Different mechanisms can contribute to the pool of intracellular α-synuclein (black arrows). Alpha-synuclein is weakly expressed in immune cells, most notably microglia, macrophages and inflammatory monocytes (panel sizes correspond to estimated relative expression). Extracellular alpha-synuclein can also contribute to the intracellular pool after uptake. Extracellular alpha-synuclein can induce the immune response by binding to molecular pattern receptors on the cell surface or the lumen of endocytic vesicles (red). Different putative mechanisms can contribute to the effects of intracellular alpha-synuclein on immune cell function (blue arrows). NK, natural killer cells; ROS, reactive oxygen species; SNCA, alpha-synuclein gene.

Alpha-synuclein, the protein central to the pathology of several neurodegenerative diseases, is well-conserved in mammals and expressed in many different tissues and cell types besides neurons of the central nervous system (CNS) (Shin et al., 2000). Although its function in the cell remains unclear – or rather not precisely defined – its effects on a plethora of cell functions have been described in detail. They include a role in synaptic plasticity, vesicle organization and release, neurotransmitter release, chaperone functions, membrane regulation and even regulation of gene expression (Emamzadeh, 2016; Surguchev and Surguchov, 2017). Almost all of this knowledge has been generated with focus on neurons and oligodendrocytes, which are the cell types mostly affected in synucleinopathies. However, the fact that alpha-synuclein is well-conserved (George, 2002), expressed in immune cells (Shin et al., 2000) and important for hematopoiesis (Xiao et al., 2014) suggests that its functions reach well beyond CNS neurons and oligodendrocytes. Similarly to those cell types, immune cells may be affected by a loss of function and/or a gain of toxic mechanism that are well-described for different pathologic manifestations of alpha-synuclein: increased concentrations of the protein, mutant forms, low- and high-molecular oligomers, large aggregates, protein fragments and post-translationally modified forms. Indeed, the discovery that alpha-synuclein and its pathologic forms are released into the extracellular space has led to an increased attention to its effects on surrounding immune cells (Ferreira and Romero-Ramos, 2018). The investigation of immune activation by extracellular alpha-synuclein now spans almost two decades of intense research and has provided very valuable insights into the mechanisms that drive and modulate neuroinflammation and cell-to-cell spreading of alpha-synuclein pathology. The recognition and processing of extracellular alpha-synuclein by immune cells can trigger their activation, proliferation, secretion of cytokines and other immune mediators, and phagocytosis (Figure 1, in red). However, very few studies have focused on the effects of *intracellular* alpha-synuclein (and its pathologic forms) on immune cells. Several observations suggest that such effects may take place and be relevant for disease: (i) some studies have shown that the local environment may be at least partly responsible for alpha-synuclein pathology, which can therefore take place also in immune cells (Candelise et al., 2020) (ii) genetic aberrations such as point mutations, duplications, and triplications are systemic and found also in immune cells (Gardai et al., 2013; Haenseler et al., 2017) (iii) alpha-synuclein is capable of escaping into the intracellular space after uptake by cells (Freeman et al., 2013; Flavin et al., 2017). The detailed study of the effects of intracellular alpha-synuclein on immune cells bears potential in two main aspects: It may reveal so-far-overseen mechanisms of neuroinflammation, and can hold diagnostic value, as peripheral immune cells are easily accessible.

Two main categories of immune cells can be distinguished in the context of synucleinopathies: CNS and peripheral immune cells. CNS immune cells comprise mainly microglia, with some functional overlap to astrocytes. While the contributions of these cell types to neurodegeneration is widely described, peripheral immune cells have just recently emerged as contributors to degenerative processes, rather than just passive bystanders. In Parkinson’s disease, a role for monocytes and T cells have recently received strong support by findings in animal models and the human disease (Harms et al., 2017a,b; Sulzer et al., 2017; Sommer et al., 2018; Grozdanov et al., 2019; Lindestam Arlehamn et al., 2020).

## Intracellular Alpha-Synuclein Pool

Two main mechanisms can contribute to the pool of intracellular alpha-synuclein: expression and uptake from the extracellular space (Figure 1, in black). Compared to neuronal cells, the expression in non-neuronal CNS cells is relatively low (see e.g., www.brainrnaseq.org). In mice, the expression in neurons is >10-fold higher than in all other brain cell types, except for oligodendrocyte precursor cells (Tabula Muris Consortium et al., 2018; Li et al., 2019). In human, single-cell transcriptomics show that microglia and brain macrophages express detectable levels of alpha-synuclein, however, still much lower than neurons and oligodendrocytes (Zhang et al., 2016). In peripheral immune cells, expression of alpha-synuclein is highest in classical monocytes and non-classical monocytes and almost negligible in T cells, B cells and other immune cell types (see e.g., https://dice-database.org/). Interestingly, expression of alpha-synuclein is relatively strong in hematopoietic precursor cells and mature erythrocytes, suggesting a role in hematopoiesis and differentiation (see below). The relative levels of alpha-synuclein expression in microglia and peripheral immune cells are hard to compare directly, but it appears that expression is stronger in inflammatory monocytes (Zhang et al., 2016; Li et al., 2019). An increased expression in microglia cannot be deduced from the expression in monocytes, as these cells behave differently in the CNS *in vivo* (Yamasaki et al., 2014). Apart from expression, intracellular alpha-synuclein can also originate from the extracellular space. Different routes of uptake have been described, including passive entry, pinocytosis and phagocytosis (for extensive review, see Tyson et al., 2016 and Grozdanov and Danzer, 2018). Aggregated alpha-synuclein can escape lyso-phagosomal compartments by vesicle rupture and gain access to the intracellular space (Freeman et al., 2013). Intracellular alpha-synuclein can affect the function of immune cells over several putative mechanisms (Figure 1, blue), e.g., generation of reactive oxygen species, modulation of gene expression, modulation of vesicle dynamics, and accumulation at the phagocytic cup.

## Intracellular Alpha-Synuclein and Microglia Function

Main functions of microglia include phagocytosis of extracellular material and apoptotic cells, surveillance and maintenance of the extracellular space, synaptic pruning, fighting off pathogens, recruitment and coordination of peripheral immune cells, self-renewal and regeneration (Li and Barres, 2018). Almost all of these functions have been shown to be affected by alpha-synuclein. Studying intracellular alpha-synuclein in microglia has been limited by two main factors: first, primary microglial cells, and to some extent microglial cell lines, are relatively hard to transfect and mostly react unspecific to the genetic manipulation, so that specific effects of the transgene are hard to differentiate. Second, most transgenic models *in vivo* target specifically neurons and do not manipulate alpha-synuclein expression in microglia. This obstacle can be overcome by the employment of universal promoters, alpha-synuclein’s own promoter or by global knockouts. Gardai et al. (2013) employed a bacterial artificial chromosome (BAC) model with overexpression of wild-type and mutant (E46K) alpha-synuclein under its own promoter and surrounding regulatory regions in mice. Purified microglia from these transgenic animals secreted significantly lower amounts of IL-6 and TNF-α despite similar endogenous expression, an effect attributed to impaired vesicle dynamics. Furthermore, isolated microglia and peritoneal macrophages displayed markedly reduced *in vitro* and *in vivo* phagocytosis of latex beads, red blood cells and apoptotic cells, which could also be linked to impaired membrane traffic and recruitment of alpha-synuclein to the phagocytic cup (Gardai et al., 2013). Further insights come from a series of studies by Combs and colleagues, who investigated the effects of alpha-synuclein knockout and overexpression on microglia (Austin et al., 2006, 2011; Rojanathammanee et al., 2011). Purified postnatal microglia from Snca^–/–^ mice presented with reactive morphology, increased basal and induced release of IL-6 and TNF-α, and a severe deficit in phagocytosis of *E. coli* bioparticles (Austin et al., 2006). Further changes in Snca^–/–^ microglia included increased levels of phospholipase D (PLD2), cytosolic phospholipase (cPLA_2_), Cox-2 and prostaglandins PGD_2_ and PGE_2_ and, importantly, increased neurotoxicity in a co-culture system (Austin et al., 2011). In a complementary approach with the murine BV-2 microglial line, transient overexpression of wild-type and mutant (A30P, A53T) human alpha-synuclein resulted in similar changes in microglia: increased levels of Cox-2, decreased phagocytosis and reduced lysosomal protein expression, but increased basal and LPS-induced IL-6, TNF-α, and NO-release. In contrast to Snca^–/–^ microglia, BV-2 cells overexpressing wild-type and mutant alpha-synuclein were not neurotoxic and did not display elevated levels of PLD2 and cPLA_2_ (Rojanathammanee et al., 2011). Interestingly, the authors observed an accumulation not only of monomeric alpha-synuclein, but also of SDS-resistant oligomeric species. Another study by Kim et al. (2009) demonstrated increased expression of CD44 and MT1-MMP by BV-2 cells transiently overexpressing human alpha-synuclein (wild-type, A30P, A53T), as well as increased migration upon overexpression of A53T. These findings clearly demonstrate that pathologic alterations in alpha synuclein interfere with murine microglial function. However, it cannot be ruled out that the observed changes in the microglial phenotype with alpha-synuclein overexpression do not result from the endogenous alpha-synuclein, but from the exposure to extracellular alpha-synuclein released from microglia and other cell types. Indeed, Haenseler et al. (2017) have demonstrated that conditioning of iPSC-derived microglia (pMac) with exogenous alpha-synuclein decreases phagocytosis in the same fashion as increased endogenous levels, while Kim et al. (2009) demonstrated the same effects of intracellular and extracellular alpha-synuclein on microglial migration.

Studies with human microglia are largely absent, but critical for the translation of insights generated with animal models. The major hurdle comes from the difficulties in obtaining and maintenance of human microglia and the limitations of cell lines (Stansley et al., 2012). However, recent advancements with the generation of microglia from peripheral blood monocytes (Etemad et al., 2012) and iPSCs (Haenseler et al., 2017) may prove useful in overcoming these limitations. Haenseler et al. (2017) generated PSC-macrophages (pMac) from PD patients with A53T mutation or triplication of alpha-synuclein, which are highly similar to brain-resident microglia. pMac from alpha-synuclein triplication, but not A53T mutation, showed increased intracellular levels of alpha-synuclein and associated reduction in phagocytosis activity and release of CXCL1, IL-18, and IL-22 (Haenseler et al., 2017). However, caution is warranted when translating findings from monocyte-derived cells to microglia, as these cells have differential roles in the CNS in health and disease (Yamasaki et al., 2014).

## Intracellular Alpha-Synuclein and Peripheral Immune Cell Function

Contrary to microglia studies, insights about intracellular alpha-synuclein on peripheral immune cell function stem mostly from human studies, due to the limited availability of human microglia and rodent peripheral immune cells. Such studies are further complicated by the difficulties in genetic manipulation and maintenance of primary cells and the therefrom-derived cell lines. Tashkandi et al. (2018) reported ultrastructural changes in peripheral blood leukocytes from a Snca^–/–^ mouse model. The findings included changes in size and shape of secretory particles, increase in smooth-endoplasmic reticulum, specific granules and inclusions (Tashkandi et al., 2018). Maitta et al. (2011) have further demonstrated a critical role for alpha-synuclein in hematopoiesis using an alpha-synuclein knockout model. Snca^–/–^ mice displayed anemia, smaller platelets, reduced B-cell maturation and defects in the generation of IgG, but not IgM (Xiao et al., 2014), as well as impaired maturation of T cells and significant reduction of total T cells (Shameli et al., 2016). Furthermore, T cells were hyperactive and released increased levels of IL-2 and decreased levels of IL-4. Together, these studies strongly suggest a critical role for alpha-synuclein in hematopoiesis. Interestingly, alpha-synuclein expression has been linked not only to physiological hematopoiesis, but also to neoplastic conditions (Maitta et al., 2011).

A valuable tool for human studies is provided by cases of familial disease, as exemplified by numerous studies with patients with a LRRK2 mutation (Speidel et al., 2016). However, alpha-synuclein-linked familial PD is very rare compared to LRRK2 familial PD (Klein and Westenberger, 2012) and only a few studies have been conducted. Gardai et al. (2013) demonstrated that the deficit in phagocytosis found in microglia from transgenic mice are reflected by fibroblasts and peripheral blood monocytes from a PD patient with triplication of the alpha-synuclein gene (SNCA) and increased intracellular levels of alpha-synuclein. Moreover, the same was also observed in fibroblasts and PBMCs from a cohort of patients with sporadic PD, where phagocytosis ability correlated negatively with intracellular levels of alpha-synuclein (Gardai et al., 2013). We have previously also reported a decrease of phagocytosis in peripheral blood monocytes from PD patients, but did not investigate alpha-synuclein levels (Grozdanov et al., 2014). A further study on PBMCs from PD patients confirmed increased expression of alpha-synuclein and showed increased propensity for apoptosis, increased expression of glucocorticoid receptor, activation of caspase-8 and caspase-9, upregulation of CD95 and increased production of reactive oxygen species (Kim et al., 2004).

## Discussion

The studies described above utilize different approaches to investigate alpha-synucleins’ role in immune cell function. Interestingly, loss of alpha-synuclein and overexpression/mutations show similar detrimental effects on immune cell function, similarly to the effects observed in other cell types. These observations suggest that the impairment in immune cell function result at least partially from a loss of function of intracellular alpha-synuclein. Furthermore, Gardai et al. (2013) demonstrated that the effect of alpha-synuclein on phagocytosis in postnatal microglia is direct and not the result of a developmental failure. It remains almost unexplored whether alpha-synuclein can aggregate also in microglia and what contribution microglial alpha-synuclein has to the initiation and spread of aggregation. Such studies are hindered by the inability to differentiate between alpha-synuclein originating from microglia or endocytosed from the extracellular space. We have recently developed an *in vivo* model which utilizes bimolecular fluorescence complementation and demonstrated the spread of neuronal alpha-synuclein from cell to cell (Kiechle et al., 2019). Such models could be modified with the use of microglia-specific promoters to investigate the dynamics of microglial alpha-synuclein and its aggregation. A further interesting paradigm emerges from the relation between inflammatory stimulation and alpha-synuclein expression. Several studies have suggested increased expression of alpha-synuclein in macrophages and astrocytes (Tanji et al., 2001, 2002) after stimulation with lipopolysaccharide and interleukins. However, it remains uninvestigated if such an increase in alpha-synuclein expression can contribute to the aggregation and cell-to-cell spread. If confirmed, this could provide a link between alpha-synuclein pathology and the association of PD with inflammatory processes. Many studies have addressed neuroinflammation in animal models of synucleinopathies, and some of them have utilized systemic, not neuron-limited approaches (Cicchetti et al., 2002; Qin et al., 2007; Gao et al., 2011). However, the insights about endogenous intracellular alpha-synuclein effects on immune cells from the latter are hard to distinguish from the effects of extracellular alpha-synuclein expressed and released by other cell types. As much as this is an obstacle, it also presents as an opportunity, as isolated effects of intracellular alpha-synuclein on immune cells may have remained so far obscured from our attention.

The deficits in phagocytosis observed in the disease models and patients with PD are, in contrast to activation and cytokine release, clearly detrimental and can be addressed therapeutically. McConlogue and colleagues have developed small molecule drug-like compounds, which effectively inhibit alpha-synuclein aggregation and recruitment to the phagocytic cup and employed them to overcome phagocytosis deficits resulting from alpha-synuclein overexpression (Toth et al., 2014, 2019). Such compounds are more appropriate for targeting intracellular alpha-synuclein than antibodies that target mostly (but not exclusively) extracellular alpha-synuclein and over therapies directed against alpha-synuclein expression, which may interfere with its physiological functions. The measurement of total alpha-synuclein in peripheral immune cells emerges as a promising diagnostic tool (Kim et al., 2004; Gardai et al., 2013; Limgala and Goker-Alpan, 2019). Human white blood cells are easily accessible, can be obtained as parallel samples for validation and longitudinally over long periods. However, measuring alpha-synuclein levels in white blood cells and its diagnostic potential has to be validated in different centers and settings, and most importantly, specificity and sensitivity have to be determined. In conclusion, the dissection of intracellular alpha-synucleins’ effects on immune cell function offers several interesting diagnostic and therapeutic possibilities and will deepen our understanding of the complex cellular events that lead to neurodegenerative diseases.

## Author Contributions

VG and KMD designed and wrote the manuscript. Both authors contributed to the article and approved the submitted version.

## Conflict of Interest

The authors declare that the research was conducted in the absence of any commercial or financial relationships that could be construed as a potential conflict of interest.
